# Ethanol Induces Secretion of Proinflammatory Extracellular Vesicles That Inhibit Adult Hippocampal Neurogenesis Through G9a/GLP-Epigenetic Signaling

**DOI:** 10.3389/fimmu.2022.866073

**Published:** 2022-05-13

**Authors:** Jian Zou, T. Jordan Walter, Alexandra Barnett, Aaron Rohlman, Fulton T. Crews, Leon G. Coleman

**Affiliations:** ^1^ Bowles Center for Alcohol Studies, University of North Carolina at Chapel Hill School of Medicine, Chapel Hill, NC, United States; ^2^ Department of Pharmacology, University of North Carolina at Chapel Hill School of Medicine, Chapel Hill, NC, United States; ^3^ Department of Psychiatry, University of North Carolina at Chapel Hill School of Medicine, Chapel Hill, NC, United States

**Keywords:** alcohol, inflammation, neurogenesis, microglia, epigenetics, G9a

## Abstract

Adult hippocampal neurogenesis (AHN) is involved in learning and memory as well as regulation of mood. Binge ethanol reduces AHN, though the mechanism is unknown. Microglia in the neurogenic niche are important regulators of AHN, and ethanol promotes proinflammatory microglia activation. We recently reported that extracellular vesicles (EVs) mediate ethanol-induced inflammatory signaling in microglia. Therefore, we investigated the role of EVs in ethanol-induced loss of adult hippocampal neurogenesis. At rest, microglia promoted neurogenesis through the secretion of pro-neurogenic extracellular vesicles (pn-EVs). Depletion of microglia using colony-stimulating factor 1 receptor (CSFR1) inhibition *in vivo* or using *ex vivo* organotypic brain slice cultures (OBSCs) caused a 30% and 56% loss of neurogenesis in the dentate, respectively, as measured by immunohistochemistry for doublecortin (DCX). Likewise, chemogenetic inhibition of microglia using a CD68.hM4di construct caused a 77% loss in OBSC, indicating a pro-neurogenic resting microglial phenotype. EVs from control OBSC were pro-neurogenic (pn-EVs), enhancing neurogenesis when transferred to other naive OBSC and restoring neurogenesis in microglia-depleted cultures. Ethanol inhibited neurogenesis and caused secretion of proinflammatory EVs (EtOH-EVs). EtOH-EVs reduced hippocampal neurogenesis in naïve OBSC by levels similar to ethanol. Neurogenesis involves complex regulation of chromatin structure that could involve EV signaling. Accordingly, EtOH-EVs were found to be enriched with mRNA for the euchromatin histone lysine methyltransferase (Ehm2t/G9a), an enzyme that reduces chromatin accessibility through histone-3 lysine-9 di-methylation (H3K9me2). EtOH-EVs induced G9a and H3K9me2 by 2-fold relative to pn-EVs in naïve OBSCs. Pharmacological inhibition of G9a with either BIX-01294 or UNC0642 prevented loss of neurogenesis caused by both EtOH and EtOH-EVs. Thus, this work finds that proinflammatory EtOH-EVs promote the loss of adult hippocampal neurogenesis through G9a-mediated epigenetic modification of chromatin structure.

## 1 Introduction

Heavy alcohol use and alcohol use disorder (AUD) are significant causes of morbidity worldwide. Chronic alcohol (i.e., ethanol) abuse causes dysfunction of key behaviors that contribute to ongoing misuse and age-related functional decline such as depressed mood, cognitive decline, and deficits in learning and memory plasticity. Adult hippocampal neurogenesis (AHN) occurs in the dentate gyrus and plays an important role in each of these functions (1–3). Chronic binge alcohol intake causes a loss of AHN with corresponding behavioral deficits (4–8). However, the underlying cause of the ethanol-induced loss of AHN is unknown.

AHN requires cooperation of multiple cell types and epigenetic regulation of specific transcriptomic cassettes (9–12). Proinflammatory activation of microglia, the resident monocyte/macrophage in brain, blunts AHN in infection or stress models (13–17). However, recently, resting microglia have been found to promote AHN through their secretome (18–20). Therefore, insults that modulate microglial activation state can regulate neurogenesis. Binge levels of ethanol promote proinflammatory microglial activation (21–23). Interventions that reduce induction of proinflammatory cytokines in brain, such as indomethacin and exercise, protect against ethanol inhibition of AHN (24). Therefore, we hypothesized that loss of AHN caused by ethanol involves secretion of anti-neurogenic factors from proinflammatory microglia. However, it is unknown how secreted factors from microglia alter transcriptional profiles in neuro-progenitors, to result in loss of AHN. In both embryonic and adult stages, epigenetic modifications of histone chromatin structure (e.g., acetylation and methylation) regulate neurogenesis (10–12, 25–28). Several enzymes are capable of remodeling chromatin structure. Several enzymes are capable of remodeling chromatin structure. We focused on the euchromatin histone-methyl transferase (EHMT2/G9a) since it has been implicated in facilitating changes in neuronal phenotype in response to ethanol (29, 30). G9a forms an enzymatic complex with a highly homologous protein G9a-like protein (GLP) to promote monomethylation and dimethylation of the histone 3 lysine 9 locus (H3K9me and H3K9me2 respectively), to play key roles in neurodevelopment (12). G9a is increased by ethanol, and alters neuron-specific genes in the basal forebrain (29, 30). Further, H3K9 methylation status is thought to regulate to neural cell proliferation (28). Therefore, we hypothesized that the regulation of AHN by ethanol and microglia involves secretion of EVs capable of regulating G9a/GLP-mediated epigenetic modifications of chromatin structure.

We recently reported that ethanol induction of proinflammatory signaling in brain involves the secretion of extracellular vesicles (EVs) from microglia (31). EVs are small, lipid-bilayer particles released from virtually all cell types that can affect the function of target cells (32). These proinflammatory EtOH-EVs were produced by microglia and reproduced the induction of proinflammatory cytokines caused by ethanol, and blocking their secretion prevented cytokine induction caused by ethanol. EVs have emerged as key mediators of disease pathology across organ systems (32–34). EVs facilitate the transfer of diverse cargo (i.e., protein, lipid, mRNA, miRNA, lncRNA, DNA) to target cells and/or elicit responses through interaction with cell surface receptors. EVs may cause epigenetic modifications to chromatin and DNA (35). In the context of ethanol exposure, EVs may participate in mediating transgenerational epigenetic inheritance (36) and regulating fetal neural stem cell development (37). Therefore, we hypothesized that proinflammatory EVs secreted in response to ethanol regulate AHN through epigenetic mechanisms. To study mechanisms regulating AHN we used both *in vivo* and *ex vivo* primary organotypic brain slice (OBSC) experiments. OBSC has all the brain cellular components and extracellular matrix of the adult hippocampal neurogenic niche and matures *ex vivo* to a neurological phenotype consistent with young adulthood with mature synapses and ongoing AHN (38–42). The use of OBSCs allows for EV transfer experiments to specifically identify the role of EVs. We found that resting microglia promote AHN through the secretion of pro-neurogenic (pn) EVs. EVs secreted by ethanol (EtOH-EVs) were proinflammatory and blunted AHN through a euchromatic histone-methyl transferase (EHMT2/G9a) epigenetic mechanism.

## 2 Materials and Methods

### 2.1 Animals

Adult C57BL/6 mice were obtained from Jackson Laboratory. Pregnant Sprague Dawley rat mothers from Charles River (Raleigh, NC, USA) were used for all slice culture preparation. All protocols followed in this study were approved by the Institutional Animal Care and Use Committee at the University of North Carolina at Chapel Hill (protocols 20-231.0 and 21-224.0) and were in accordance with National Institutes of Health regulations for the care and use of animals in research.

### 2.2 Materials

The CSF1R inhibitor PLX-3397 was obtained from MCE (Monmouth Junction, NJ, USA). PLX-5622 was provided by Plexxikon (Berkeley, CA, USA). Antibodies were purchased from the following vendors: DCX (Santa Cruz, sc-271390, USA), Ki67 (abcam, ab16667, Boston, USA), G9a (abcam, ab185050, Boston, USA), H3K9me (abcam, ab32521, Boston, MA, USA), beta actin (abcam, ab8229). The G9a inhibitor BIX-01294 was purchased from Selleckchem (Houston, TX, USA) and UNC0642 is from Santa Cruz (Santa Cruz, CA, USA).

### 2.3 Microglial Depletion *In Vivo*

Microglia were depleted brain-wide using the colony stimulating factor 1 receptor (CSF1R) antagonist PLX-5622 supplemented in chow (1200 ppm) as described previously (43). Briefly, mice were given PLX-supplemented or normal chow for three weeks. Mice were sacrificed by intracardial perfusion with paraformaldehyde, and brains processed for immunohistochemistry (IHC) as we have reported previously (43, 44).

### 2.4 Primary Organotypic Brain Slice Culture (OBSC)

Primary organotypic brain slice cultures (OBSCs) were prepared from the hippocampal-entorhinal cortex formation of postnatal day (P)7 pups using previously established techniques of Stoppini and colleagues (45) with modifications as we have described previously (31, 46, 47). Briefly, a Sprague Dawley rat mother with a litter of 12 pups on P6 were obtained from Charles River (Raleigh, NC, USA) and housed overnight in the animal facility. All neonates at P7 were decapitated, the brains were removed, and hippocampal-entorhinal complex dissected in Gey’s buffer (Sigma-Aldrich, St. Louis, MO, USA). Slices (~25/pup) were transversely cut with a McIlwain tissue chopper at a thickness of 375 µm and placed onto a 30 mm diameter Millicell low height culture insert (Millipore, PICMORG50), 10-13 slices/tissue insert. Slices from both sexes were pooled together. Slices were cultured with MEM containing 25 mM HEPES and Hank’s salts, supplemented with 25% horse serum (HS) + 5.5 g/L glucose + 2 mM L-glutamine in a humidified 5% CO2 incubator at 36.5°C for 7 days *in vitro* (DIV), followed by 4DIV in MEM + 12% HS, and then slices were cultured with MEM + 6% HS until the end of experiments. Two replicates were performed for each experimental condition, with each experiment repeated at least twice. Serum-free N2-supplemented MEM was used to mix with MEM containing 25% HS throughout experiments. OBSC slices were incubated for a total of 14 days prior to treatment as described by Stoppini et al. to allow for functional maturation of synapses (45). OBSC slices are long-lived with undetectable cell death up to 42 days in culture in our previous report (21).

For transfection of the hM4di DREADD in microglia, OBSCs were treated with either control AAV9.EGFP or AAV9.CD68.hm4Di.mCherry (VectorBuilder) for 24 h prior to administration of the hM4di agonist CNO for 4 days.

### 2.5 Microglial Depletion in OBSC

For microglial depletion in OBSC, slices at 4DIV were treated with the CSF1R inhibitor PLX-3397 (1µM), chosen for solubility in media, for 7 days in regular culture medium (MEM containing 25% HS), followed by 3DIV in MEM + 12% HS to deplete microglia. We used 1µM PLX-3397 since we have previously reported that this successfully depletes >90% of microglia in our *ex vivo* slice culture model (21). At the end of PLX3397 treatment, slices were either immediately removed for analysis or followed by microglial repopulation protocol in MEM + 6%HS without PLX-3397 (21).

### 2.6 Immunohistochemistry (IHC) and Quantification *In Vivo* and in OBSC

#### 2.6.1 *In Vivo*:

IHC was performed on PFA-perfused mice as we have reported previously (43). Briefly, perfused brains were dehydrated in 30% sucrose. Brains were then sliced at 40µM thickness using a freezing microtome. Free-floating sections were washed in 0.1M PBS, endogenous peroxidases neutralized by incubation in 0.3% H_2_O_2_ for 30 min, washed again in PBS, and permeabilized and blocked for 1 h in 0.25% Triton-X100/5% normal serum at room temperature (RT). Sections were then incubated overnight at 4°C in primary antibody in blocking solution. The next day sections were washed in PBS and incubated in biotinylated secondary antibody (1:200; Vector Laboratories, Burlingame, CA, USA) for 1 h at RT. After washes sections were incubated in avidin–biotin complex solution (Vectastain ABC Kit, Vector Laboratories, Burlingame, CA, Cat. # PK6100) for 1 h at RT. Immunoreactivity was visualized using nickel-enhanced diaminobenzidine (Sigma-Aldrich, St. Louis, MO, USA, Cat. # D5637). Tissue was mounted onto slides, dehydrated, and coverslips placed. At least three sections per mouse were quantified for immunoreactivity (+IR)

#### 2.6.2 *OBSC:*

At the end of each experiment, OBSC slices were quickly washed in cold PBS and fixed with 4% paraformaldehyde in 0.1M PBS for 24 h at 4°C. Floating OBSC slices were washed with PBS and incubated with 0.6% H_2_O_2_ to inhibit endogenous peroxidase. After further washing with PBS, slices were blocked with 3% rabbit serum containing 0.25% Triton X100 for 1 h at RT and then incubated with mouse anti-DCX or anti-H3K9me2 for 48 h at 4°C. DCX+IR was detected using an ABC kit and followed by the DAB method. For quantification of DCX IR, DCX+ cell and processes in DG region of the hippocampus were imaged live at 8x magnification, background corrected, and DCX IR measured with the Keynce BZ-H4XF imaging software (*in vivo*) or ImageJ (OBSC).

### 2.7 EV Isolation and Assessment by Nanoparticle Tracking Analysis and Transmission Electron Microscopy (TEM)

#### 2.7.1 EV Isolation:

EVs were isolated by sequential centrifugation from OBSC media as we have reported previously (31, 48). Briefly, media were centrifuged at 300 g for 10 min and followed by 6000g for 10 min to remove cellular debris. The supernatant was then centrifuged at 21,000g for 96 min at 4°C to pellet EVs. This method isolates primarily microvesicles (MVs, 0.05-1.0µm diameter) and larger exosomes (0.05-0.1µm diameter) (32). EVs were resuspended in fresh MEM media.

#### 2.7.2 *TEM Negative Stain*:

EVs were visualized by negative-stain transmission electron microscopy (TEM) in the UNC Microscopy Services Laboratory. Briefly, this involved floating a glow-discharged formvar/carbon-coated 400 mesh copper grid (Ted Pella, Inc., Redding, CA, USA) on a 20μl droplet of the sample suspension for 12 min. This was transferred quickly to 2 drops of deionized water followed by addition of a droplet of 2% aqueous uranyl acetate stain for 1 minute. The grid was blotted with filter paper and airdried. Samples were then visualized at 80kV using a JEOL JEM-1230 transmission electron microscope operating (JEOL USA INC., Peabody, MA, USA). Images were taken using a Gatan Orius SC1000 CCD camera with Gatan Microscopy Suite version 3.10.1002.0 software (Gatan, Inc., Pleasanton, CA, USA).

#### 2.7.3 *Nanoparticle Tracking Analysis (NTA)*:

Media samples were diluted in 0.02 µm filtered PBS and assessed by NTA on the ParticleMetrix ZetaView^®^ PMX-420 Video Microscope in the Nanomedicines Characterization Core Facility at UNC as we have reported previously (31, 49–51).

### 2.8 Ethanol Treatment and Conditioned EV Transfers in OBSC

All ethanol treatments with the indicated concentrations occurred in a desiccator containing 300ml water plus ethanol at the same concentration present in the culture media as we have reported previously (31, 52, 53). OBSC slices at 14DIV were exposed to ethanol (100mM) for 4 days in the absence or presence of G9a inhibitor BIX-01294. We have previously found this concentration *ex vivo* causes induction of immune genes that is similar to findings *in vivo* and in postmortem human AUD brain (48, 53). Though this is a high concentration of alcohol for non-dependent individuals, patients with AUD reach these blood alcohol concentrations while being conscious and functional (54). At the end of experiments, slices were removed for further analysis.

For conditioned EV transfer experiments, pelleted EVs from control or ethanol-treated groups were resuspended in MEM + 6% horse serum. This media and serum concentration has been optimized in our previous studies for the assessment of neurogenesis in the OBSC (42). To account for potential effects of EVs in the media, all experiments include unconditioned media controls as recommended by the International Society for Extracellular Vesicles (ISEV) guidelines in situations when the use of exosome-depleted media is not desirable (55). We also perform EV supernatant controls (described below). We have reported that this technique recovers ~30% of total media MVs, thus a ratio of EVs pelleted from 2 slice culture wells to 1 new slice culture well is transferred (31). EVs were isolated from 1 mL of culture media. Each culture well had 10-13 hippocampal slices (300µm thickness). 1.4x10^10^ EVs were added to each recipient culture which is ~20% of the total slice media EVs at baseline, similar to our previous studies (31, 49, 50). At the end of EV treatment, slices were removed for either mRNA analysis by RT-PCR or immunostaining for measurement of neurogenesis. To determine if remaining proteins in the EV preparations mediate the effects of EVs two methods were employed. First, supernatant (SUPN) from the final spin of EV isolation was transferred to naïve OBSC as we had done previously (31) to determine if soluble components in media mediate the effects as recommended by the ISEV guidelines (55). Briefly, SUPN from the final spin was diluted 1:1 with fresh media to ensure adequate nutrition and was then added to naive OBSC. Further, we also performed digestion of remaining proteins in the EV preparation using proteinase K as described previously (56). Briefly, pelleted EVs were resuspended in PBS and incubated with 20µg/mL of Proteinase K (Prot K, Invitrogen) at 37°C for 1 hour. Prot K activity was quenched by adding 5mM phenylmethylsulfonyl fluoride (10 min at RT). EVs were then re-pelleted (21,000g, 96 minutes) and resuspended in fresh MEM, then added to naïve OBSC.

### 2.9 Measurement of mRNAs and Protein in EVs and OBSC Tissue

#### 2.9.1 RNA Isolation *and Reverse Transcription*:

Total RNA was isolated either from the pelleted EVs or OBSC slice tissue using miRNAeasy Kit (Qiagen Inc., CA, USA) according to manufacturer’s protocol. Briefly, the pelleted EVs from 2-3 culture media or 10-13 pooled tissue slices/per group were dissolved in total of 700ul Trizol lysis buffer as we have previously reported (21, 31). After centrifugation at 12,000g for 15 min, supernatant was removed for total RNA purification. The total amount of RNA was quantified by Nanodrop™. For reverse transcription, either 200ng of RNA from EVs or 2 μg of RNA from slices was used to synthesize the first strand of cDNA using random primers (Invitrogen) and reverse transcriptase Moloney murine leukemia virus (Invitrogen). After a 1:2 dilution with water, 2 μl of the first strand cDNA solution was used for RT-PCR.

#### 2.9.2 *RT-PCR*:

The primer sequences for real time RT-PCR were as follows: TNFα-F: AGCCCTGGTATGAGCCCATGTA, R:CCGGACTCCGTGATGTCTAAG; IL-1β-F:TTGTGCAAG TGTCTGAAGCA, R: TGTCAGCCTCAAAGAACAGG; G9a-F:CTCCGGTCCCTTGTCTCC, R:CT ATGAGAGGTGTCCCCCAA; β-Actin-F:CTACAATGAGCTGCGTGTGGC, R: CAGGTCCAGAC GCAGGATGGC; 18S-F: CGGGGAATCAGGGTTCGATT, R:TCGGGAGTGGGTAATTTGCG. Primer sequences were validated using the NCBI Primer-BLAST, with only primers that targeted the desired mRNA and had single-peak melt curves (showing amplification of a single product) being used. SYBER Green Supermix (AB system, UK) was used as a RT-PCR solution. The real time RT-PCR was run with initial activation for 10 min at 95°C and followed by either 48 cycles (EV mRNA) or 40 cycles (tissue mRNA) of denaturation (95°C, 40 s), annealing (58°C, 45 s) and extension (72°C, 40 s). The threshold cycle (*C*_T_) of each target product was determined and normalized to a reference housekeeping gene. For all analyses in OBSC tissue, β-actin was used as the housekeeping gene. For measurement of mRNAs in EVs, 18S was used as the housekeeping gene since actin mRNA was not detected in the vesicles. Difference in *C*_T_ values (Δ*C*_T_) of two genes was calculated $[\text{difference}=2^{-{(}_{\text{T}}^{C}\;of\;\text{target enes}-{\;}_{\text{T}}^{C}\text{ of }\beta -\text{actin})}\;=2_{\text{T}}^{-\Delta C}$ and the result was expressed as the percentage compared to control.

#### 2.9.3 *Western Blot*:

OBSC sections (4-6) were pooled and lysed with Tris buffer and centrifuged at 21,000g to remove the nuclear fraction as we previously reported (48). Protein concentrations were measured using a Pierce BCA assay kit with the manufacturer’s instructions (ThermoFisher Scientific). Samples were diluted in RIPA and DTT containing reducing buffer (Pierce TM Cat. # 39000) to a final amount of 40 μg protein per well. Protein samples were separated on a 4–15% Ready Gel Tris-HCL gel (BioRad) and transferred onto PVDF membranes (BioRad). PVDF membranes were incubated at 4°C overnight with primary antibody. The following day, secondary antibody incubation (Li-Cor IRDye^®^ 680 or 800) was performed, and membranes visualized using the LiCor Odyssey™ imaging system. Values for proteins of interest were normalized to beta-actin expression for each sample.

### 2.10 Statistical Analyses

For experiments when only two groups were compared, *t*-tests were performed. Statistical analyses for experiments with multiple treatment groups were performed using One-way ANOVAs, with Sidak’s *post hoc* tests for multiple comparisons. Differences were considered statistically significant if *p* < 0.05.

## 3 Results

### 3.1 Microglia Promote Neurogenesis Through the Secretion of Pro-Neurogenic Extracellular Vesicles (pn-EVs)

**Figure 1:**
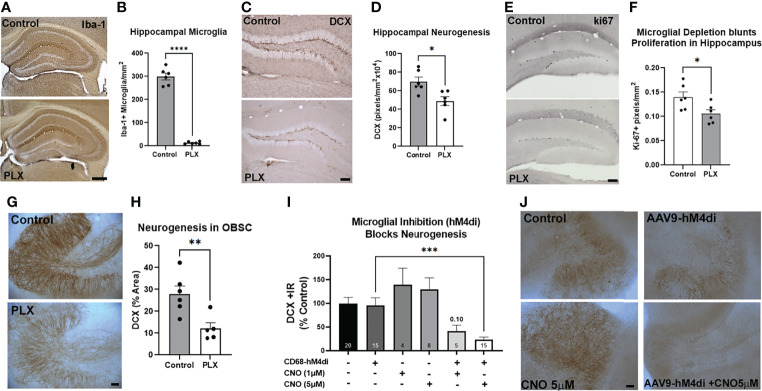
Microglia at rest promote adult hippocampal neurogenesis. **(A-F)** Adult mice were given either normal or PLX-5622-supplemented chow for 3 weeks to deplete microglia. **(A)** PLX chow caused robust depletion of microglia as measured by Iba-1 IHC. **(B)** Quantification of Iba-1 staining in hippocampus found ~96% depletion of microglia. ****p <  0.0001, *t-*test vs. control. Scale bar: 500µm **(C)** Representative images of DCX staining in hippocampal dentate gyrus showing loss of neurogenesis with microglial depletion. Scale bar 100 µm. **(D)** Quantification found a ~30% reduction in DCX immunoreactivity. *p <  0.05, *t-*test vs. control. **(E)** Representative images of ki67 immunostaining after EtOH in dentate regions of the hippocampus in control and microglia depleted (PLX) mice. **(F)** Ethanol caused a 24% reduction in ki67. *p < 0.05, *t-*test. **(G, H)** OBSC were treated with PLX-3397 (1µM) for 10 days to deplete microglia and determine its effect on neurogenesis. **(G)** Representative images of DCX in hippocampal region of OBSC showing loss of neurogenesis with microglial depletion. Scale bar: 100µm. **(H)** Quantification found a 56% reduction in DCX staining with microglial depletion. **p < 0.01, *t-*test vs. control. **(I, J)** Microglial inhibition reduces neurogenesis. OBSC were treated with either AAV9.CD68.hM4di or AAV9.EGFP control for 24 hours prior to treatment with CNO (1µM or 5µM) or vehicle for 4 days, followed by IHC for DCX. **(I)** Quantification of DCX found that neither viral transfection alone nor CNO alone significantly altered neurogenesis. Addition of CNO to microglial hM4di-transfected slices caused a 77% reduction in DCX with 5µM CNO. 1-Way ANOVA, F_5,61 _= 7.148, *p< * 0.0001. ***p < 0.001, Sidak’s *post-hoc* test vs. AAV9.CD68.hM4di alone **(J)** Representative images of DCX +/- CNO and AAV9 transfection. Scale bar: 100µm.

Microglia have been found to promote adult hippocampal neurogenesis through their secretome (18–20). EVs are a key aspect of the microglial secretome (31); therefore we assessed their role in neurogenesis. First, we determined the impact of microglial depletion on neurogenesis both *in vivo* and *ex vivo*. Microglia were depleted using the CSF1R antagonist PLX-5622 as reported previously (43). PLX5622 effectively removed Iba1+ microglia in the hippocampus with ~96% depletion of microglia (**Figures 1A, B**). We then assessed hippocampal neurogenesis in the dentate gyrus using immunohistochemistry (IHC) for doublecortin (DCX). DCX is a cytoskeleton marker of neuroprogenitors expressed during maturation of dendrites and other neuronal structures. DCX was reduced by 30% in microglia-depleted mice (**Figures 1C, D**, *p<0.05). Likewise, a 24% reduction in Ki67, a marker of proliferation, was found (**Figures 1E, F**). The role of microglia in neurogenesis was then assessed in OBSC, where PLX depletion of microglia caused a 56% reduction in DCX in the dentate gyrus (**Figures 1G, H**). To confirm that the loss of microglial activity underlies the loss of neurogenesis rather than factors released by dying microglia or off-target effects of CSF1R-inhibition, we next inhibited microglial activation using a chemogenetic approach. Microglial activity was inhibited by transfection with the hM4di inhibitory designer receptor exclusively activated by designer drugs (DREADD) using a viral construct (AAV9.CD68.hm4di) as we have reported previously (21). In the presence of microglial hM4di expression, a significant treatment effect across groups was found (ANOVA F_5,61 _= 7.148, ****p<0.0001) with the hM4di agonist CNO causing a 77% reduction in neurogenesis in the dentate gyrus as measured by DCX staining (**Figures 1I, J**, ***p<0.001, Sidak’s post-test). Neither hM4di transfection alone nor CNO alone significantly altered neurogenesis. Thus, microglia in healthy mouse dentate gyrus and OBSC brain slice culture promote hippocampal neurogenesis both *in vivo* and *ex vivo*. Since the microglial secretome has been implicated in hippocampal neuroprogenitor survival, we next studied the role of secreted extracellular vesicles.

**Figure 2:**
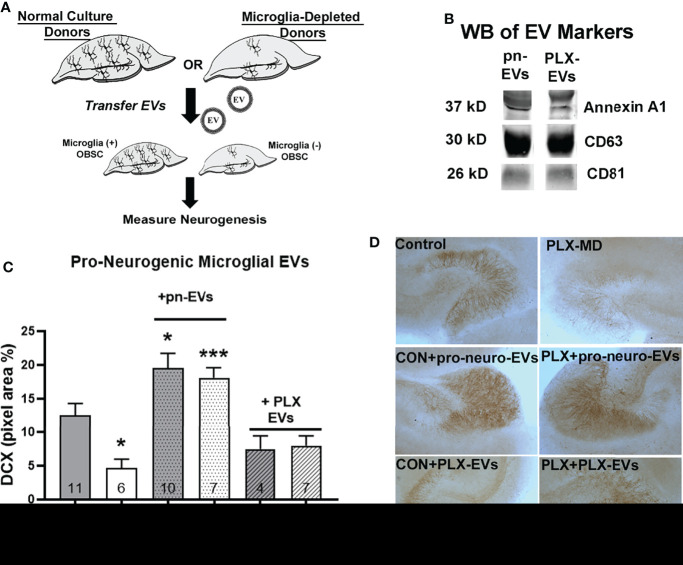
EVs from microglia-containing cultures are pro-neurogenic. **(A)** Experimental design for EV transfer experiments from normal microglia-containing and PLX microglia-depleted OBSCs. EVs from each donor condition were transferred to either microglia-containing (Control) or microglia-depleted (PLX) OBSC recipients. **(B)** Western blot analysis shows expression of EV markers Annexin A1, CD63 and CD81 in pn-EVs and PLX-EVs. **(C)** Quantification of treatment effect on DCX immunostaining (One-way ANOVA, F_5,39_ = 9.705, *p < *0.0001). Microglial depletion with PLX-3397 (1µM, 10 days) caused a 62% reduction in DCX in the dentate region of the OBSC (*p < 0.05 vs. control, Sidak’s post-test). EVs from control OBSC (pn-EVs) increased basal levels of DCX by 55% (gray bar with dots, *p < 0.05 vs. Control, Sidak’s post-test) and increased DCX in microglia-depleted recipient OBSCs by 290% (white bar with dots, ***p < 0.001 vs. PLX alone, Sidak’s post-test). EVs from microglia-depleted OBSC donors (PLX-EVs), however, did not significantly change DCX levels in control (*p=*0.43 vs. control alone, Sidak’s post-test) or microglia-depleted OBSC slices (*p=*0.78, vs. PLX alone, Sidak’s post-test) N for each group is included within each bar. **(D)** Representative images of DCX IHC in OBSC treatment groups. Scale bar 100 µm.

To determine the effect of OBSC EVs on neurogenesis, we used EV transfer experiments using our previously established protocols (31). EVs were transferred from either normal or microglia-depleted OBSC donors to either normal (microglia +) or microglia-depleted OBSC recipient cultures (**Figure 2A**). A significant treatment effect was found across the groups (F_5,39_ = 9.705, *p<*0.0001). As above, microglial depletion with PLX alone caused a robust reduction in neurogenesis as measured by DCX (62%, **Figure 2C**, *p<0.05 vs. Control, Sidak’s post-test) that was clearly visible (**Figure 2D**, top panel). EVs transferred from normal microglia-containing cultures were pro-neurogenic (pn-EVs), increasing levels of neurogenesis in normal slices by 56% (**Figures 2C, D** middle panel, *p<0.05 vs. Control, Sidak’s post-test). pn-EVs also increased the expression of the pro-neurogeneic cytokine IL-4 (57). IL-4 was increased by 82% (**Supplementary Figure 1A**, *p<0.05, Sidak’s post-test). Further, pn-EVs restored the loss of DCX in PLX microglia-depleted slices, increasing DCX by 290% relative to vehicle PLX slices (***p=0.001, vs. PLX alone, Sidak’s post-test) EVs isolated from microglia-depleted-cultures, however (**Figures 2C, D** lower panel), did not increase DCX in neither control (*p=*0.43 vs. control alone) nor microglia-depleted (*p=*0.78, vs. PLX alone) recipient OBSCs. This indicates that microglial EVs at baseline promote neurogenesis through the generation of pn-EVs.

### 3.2 Ethanol Induces Secretion of Proinflammatory EVs (EtOH-EVs) That Inhibit Hippocampal Neurogenesis

**Figure 3:**
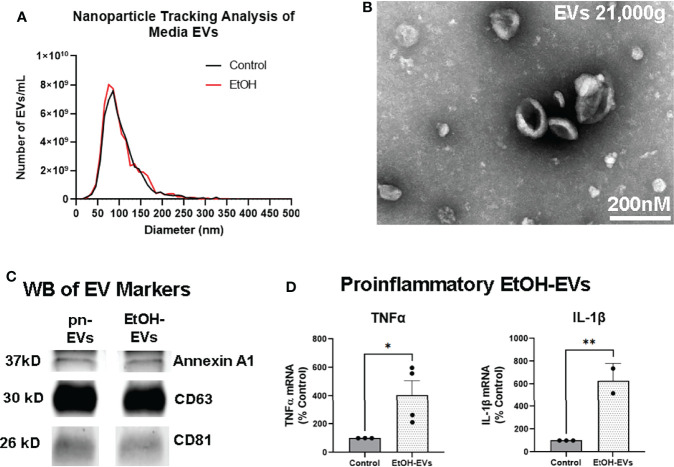
EVs secreted by ethanol are proinflammatory. EVs were isolated from OBSC media after either ethanol (EtOH-EVs) or vehicle control (pro-neurogenic, pn-EVs). **(A)** NTA assessment of OBSC media showed similar size distributions between Control/pn-EVs and EtOH-EVs, mostly between 50-300nm in diameter. Averaged distributions from 3 samples per group depicted. **(B)** Transmitting electron microscopy confirmed the presence EVs with the expected size and cup-shaped morphology. **(C)** Western blot (WB) analysis of EV markers found Annexin A1, CD63, and CD81 in both pn-EVs and EtOH-EVs. **(D)** EtOH-EVs were transferred to naïve OBSC slices, and levels of proinflammatory cytokines in slices measured by RT-PCR. A significant main effect of treatment was found (One-way ANOVA F_3,8_ = 9.211, *p=*0.0055). EtOH-EVs caused robust inductions of TNFα (4-fold) and IL-1β (6-fold), *p < 0.05, **p < 0.01 Sidak’s post-tests.

We recently reported that EVs play a key role in proinflammatory induction caused by ethanol, reproducing the effects of ethanol treatment (31). Though EtOH did not increase the number of EVs released as measured by nanoparticle tracking analysis, it altered EV contents and function (31). Proinflammatory stimuli such as binge ethanol are known to blunt neurogenesis (13, 15, 58), with the microglial secretome playing an important role in this regulation (18–20). Therefore, we investigated the role of EtOH-induced EVs on hippocampal neurogenesis. EVs were isolated from OBSC media with control or EtOH treatment as we have previously reported (31). NTA analysis confirmed EVs in the 50-300nm diameter size range in both control/pn-EVs and EtOH-EVs (**Figure 3A**). The phenotype of EVs was then assessed by TEM negative staining. TEM confirmed the presence of EVs with the expected morphology (**Figure 3B**). Western blot further confirmed the presence of EVs with positive expression of the MV marker Annexin A1, the exosomal marker CD63, and the shared MV/exosomal marker CD81 (**Figure 3C**). EtOH-conditioned EVs were proinflammatory, with adoptive transfer to naïve slices causing a significant treatment effect (**Figure 3D**, F_3,8_ = 9.211, *p=*0.0055). *Post-hoc* analyses confirmed a 4-fold increase in gene expression of TNFα (*p<0.05, Sidak’s post-test) and a 6-fold increase in IL-1β (**p<0.01 Sidak’s post-test) consistent with our previous work (31).

**Figure 4:**
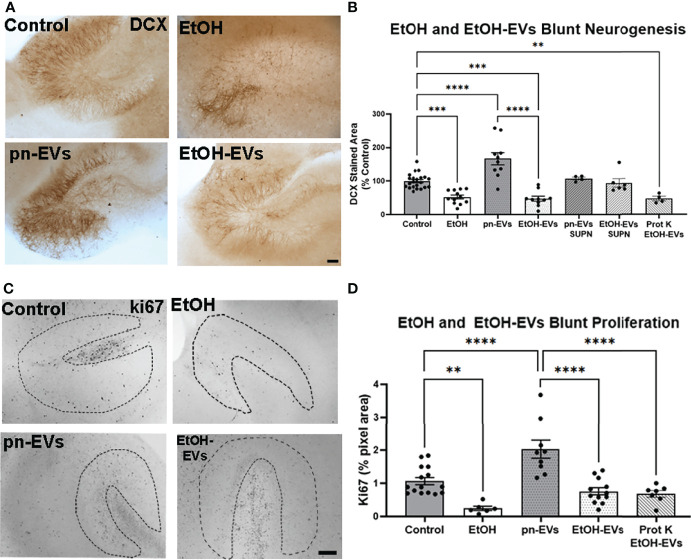
EVs secreted by ethanol blunt neurogenesis. OBSCs were treated for 4 days with either vehicle, ethanol, pn-EVs, EtOH-EVs, supernatant (SUPN) from EV preparations, or EtOH-EVs treated with proteinase K (Prot K) to degrade free proteins. Neurogenesis measured by IHC for DCX. **(A)** Representative images showing enhancement of neurogenesis by pn-EVs and a clear reduction of neurogenesis by EtOH and EtOH-EVs. Scale bar 100 µm. **(B)** Quantification of DCX staining found a significant main effect of treatment (F_5, 58_ = 20.9, p < 0.0001). A 48% reduction in DCX was caused by EtOH, a 67% increase by pn-EVs relative to control. EtOH-EVs caused a 52% reduction compared to pn-EVs. SUPN isolated from pn-EV and EtOH-EV preparations had no effect on DCX. Prot K digestion of free proteins had no effect on reduction of DCX caused by EtOH-EVs. **(C)** Representative images of ki67 immunostaining in OBSC in control, EtOH, control/pn-EVs and EtOH-EVs. The neurogenic niche in the OBSC is outlined by the dashed lines. **(D)** Quantification of ki67 found a main effect of treatment (one-way ANOVA, F_3,41 =_ 21.75, *p < *0.0001.) Ethanol-EVs caused a 45% reduction in 60% reduction in ki67 staining compared to pn-EVs. Prot K digestion free proteins had no impack on loss of Ki67 caused by EtOH-EVs.**p < 0.01, ***p < 0.001, ****p < 0.00001 Sidak’s post-test. Each dot represents an individual culture slice.

Next, we assessed the effect of EtOH and EtOH-EVs on neurogenesis. A significant main effect of treatment was found on neurogenesis as measured by DCX (**Figures 4A, B**, F_5, 58_ = 20.9, *p*<0.0001) and ki67 (**Figures 4C, D**, F_3, 22_ = 18.25, *p*<0.0001). Ethanol reduced DCX expression by 48% compared to control (**Figures 4A, B**, ***p<0.001, Sidak’s post-test), consistent with previous findings *in vivo* and *ex vivo* (6, 24, 59). EVs from control OBSC were pro-neurogenic (pn) with a 67% increase in DCX (****p<0.0001 vs. Control, Sidak’s post-test). However, EVs from EtOH-treated OBSC reduced DCX staining by ~52% compared to controls (***p<0.001) similar to the effects of ethanol. There was no significant difference between EtOH treated and EtOH-EV treated EVs (*p=*0.998). Likewise, reductions in ki67 were seen caused by ethanol (**Figure 4D**, 77%, **p<0.01 vs. control) and EtOH-EVs (**Figure 4D**, 62% vs. pn-EVs, ****p<0.0001). In order to determine if remaining media proteins in the EV preparations mediate this effect, we also assessed the effects of the EV supernatant on AHN, as recommended by the ISEV guidelines (55). This experiment is needed in order to attribute biological effects to EVs rather than a soluble component, and avoids the confounding of effects of components from size exclusion columns on biological function of recipient cells (55). We previously reported that EVs, but not EV depleted supernatant cause proinflammatory gene induction in brain slices (31). Likewise, we found that neither supernatant (SUPN) from control/pn-EV preparations, nor SUPN from EtOH-EV preparations had any effect on DCX (**Figure 4B**). Further, proteinase K digestion of EtOH-EV preparations to degrade all protein species outside of the EV membrane had no impact on the loss of DCX (**Figure 4B**) or ki67 (**Figure 4D**) caused by EtOH-EVs. This indicates that the observed effects of EtOH-EVs are not due to remaining protein in the isolated EVs. Thus, EVs secreted in response to ethanol are proinflammatory and anti-neurogenic, phenocopying the effects of ethanol. Since neurogenesis involves complex changes in chromatin accessibility to allow lineage specific gene transcription, we sought to investigate if EtOH-EV inhibition of neurogenesis involved an epigenetic mechanism.

### 3.3 EtOH-EVs Are Enriched With Euchromatin Histone Lysine Methyltransferase (Ehmt2)/G9a mRNA and Increase Histone-3 Lysine-9 Di-Methylation (H3K9me2)

**Figure 5:**
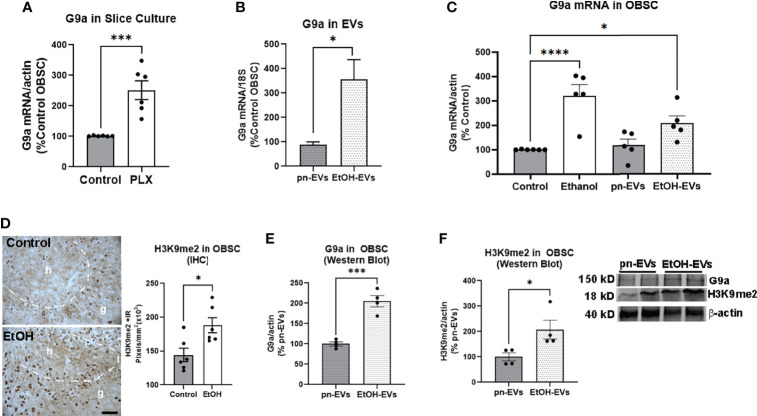
Microglia depletion and ethanol-EVs increase G9a in OBSC. **(A)** G9a mRNA was measured in control or PLX-microglial depleted OBSC. PLX resulted in a 2.5-fold increase in G9a relative to controls. N = 6 experimental replicates. ***p < 0.001, *t-*test **(B)** EVs were isolated from OBSC media after either ethanol (EtOH-EVs) or vehicle control (pn-EVs). RT-PCR of mRNA isolated from EVs found a 3.5-fold increase of G9a mRNA in EtOH-EVs. N = 2 experimental replicates/group. **(C)** G9a mRNA was increased in OBSC slice tissue after treatment with either ethanol (3-fold) or EtOH-EVs (2-fold). *p < 0.05, ****p < 0.0001, Sidak’s post-test. **(D)** IHC found EtOH increased the number of H3K9me2 immunoreactive (+IR) pixels/mm^2^ in the dentate granular (g) cell region of OBSC by 30%. The dashed white line marks the border of the hilus (h), which is the neurogenic region. Each dot represents an OBSC slice. *p < 0.05, *t-*test Scale bar 100 µm. **(E, F)** OBSCs were treated with either control (pn-EVs) or EtOH-EVs. Western blot found that EtOH EVs increased of levels of **(E)** G9a protein (2-fold) and **(F)** H3K9me2 (2.1-fold). Each dot represents 4-6 pool OBSC slices. *p < 0.05, ***p < 0.001, *t-*test. Insert with representative western blot images of G9a, H3K9me2 and actin.

Neurogenesis involves a complex orchestration of differential transcriptional programs that are regulated by chromatin modifications (10–12). EVs contain complex cargo including mRNAs, non-coding RNAs, miRNAs, and proteins that could potentially regulate epigenetic chromatin modifications that could regulate neurogenesis (33, 60). Therefore, we investigated whether EVs alter epigenetic modifications that regulate neurogenesis. The euchromatin histone lysine methyltransferase (Ehmt2/G9a) facilitates di-methylation of the lysine 9 residue on histone 3 (H3K9me2) to repress gene expression. G9a is involved in lineage specification during embryonic neurogenesis (12), and G9a-associated epigenetic marks are reduced by environmental enrichment-enhanced neurogenesis (11). G9a mRNA was increased by 2.5-fold (***p<0.001, **Figure 5A**) in microglia-depleted slices, consistent with an association between its induction and a loss of neurogenesis. In EtOH-EVs, we found that G9a mRNA was increased by more than 3-fold relative to control pn-EVs (**Figure 5B**, *p<0.05). In OBSC tissue, a main effect of treatment was found on G9a mRNA after treatment with either ethanol or EtOH-EVs (**Figure 5C**, One-way ANOVA, F_3,17_ = 12.90, *p*=0.0001). Both ethanol and EtOH-EVs increased expression of G9a mRNA by 3-fold (****p<0.0001, Sidak’s post-test) and 2-fold (*p<0.05, Sidak’s), respectively, compared to controls with no effect of pn-EVs (**Figure 5C**). Since G9a mRNA was increased in OBSC by ethanol, we assessed if its associated H3K9me2 mark was also increased. IHC revealed H3K9me2 staining primarily in the neuronal granular region of the dentate and at the neurogenic niche (**Figure 5D**, dashed line). Ethanol increased H3K9me2 in the neuronal granular region of the dentate by 30% (**Figure 5D**). We then assessed the effect of EtOH-EV treatment of OBSCs on both G9a and H3K9me2 by western blot to confirm RT-PCR and IHC findings. EtOH-EVs caused a ~2-fold increase in both G9a protein (**Figure 5E**, **p<0.01) and H3K9me2 (**Figure 5F**, **p<0.01) in OBSC tissue. Since we found induction of G9a and H3K9me2, we next investigated if pharmacological inhibition of the G9a/GLP complex could prevent the loss of neurogenesis caused by ethanol and EtOH-EVs.

### 3.4 Pharmacological Inhibition of G9a/GLP Prevents Loss of Neurogenesis by EtOH and EtOH-EVs

**Figure 6:**
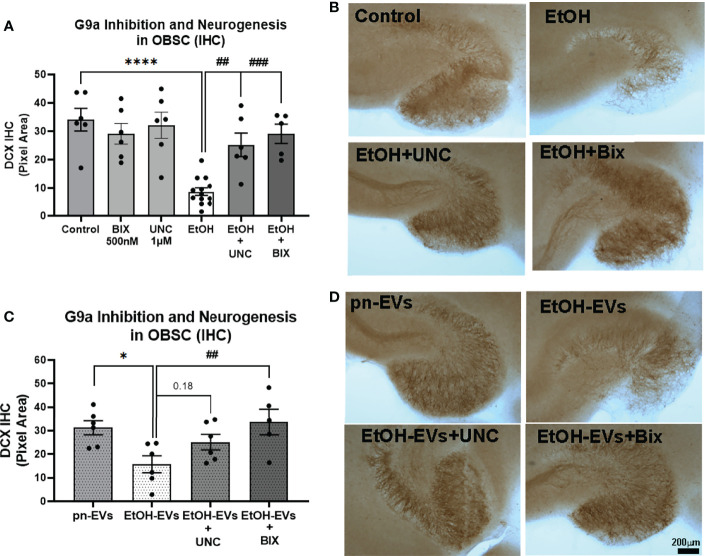
Pharmacological inhibition of G9a prevents loss of neurogenesis caused by ethanol and EtOH-EVs. OBSCs were treated with either EtOH or EtOH-EVs +/- G9a inhibitors BIX-01294 (BIX) or UNC0642 (UNC). Neurogenesis was measured by IHC for DCX. **(A)** EtOH caused a 74% reduction of DCX that was prevented by UNC (1µM) and BIX (500nM). 1-Way ANOVA, F_13,68 _= 6.8, ****p < 0.0001 vs. control, ##*p < *0.01 and *###p < *0.001 vs. EtOH, Sidak’s *post-hoc* multiple comparison’s test. **(B)** Representative images show loss of neurogenesis with EtOH that was prevented by G9a inhibitors. **(C)** pn-EVs increased DCX by 37%, while EtOH-EVs caused a 50% reduction in DCX relative to control pn-EVs. One-way ANOVA, F_4,23 _= 4.05, *p < 0.01 vs. pn-EVs, *##p < *0.01 vs. EtOH-EVs, Sidak’s post-test. **(D)** Images showing G9a inhibitors blocked the loss of neurogenesis caused by EtOH-EVs.

Since we found that ethanol and EtOH-EVs block adult hippocampal neurogenesis and increase expression of G9a, we next determined if inhibition of G9a signaling would prevent the loss of neurogenesis. We first measured G9a expression levels in OBSC after microglial depletion with PLX, which blunts neurogenesis (**Figures 1** and **2**). G9a methyl-transferase activity was then blocked using pharmacological inhibitors of the G9a/GLP complex – BIX-01294 (BIX), and UNC0642 (UNC) (61, 62). A main effect of treatment was observed (**Figure 6A**, One-way ANOVA F_13,68 _= 6.8, ****p<0.0001). As above, ethanol robustly inhibited hippocampal neurogenesis in OBSC (74% reduction, **Figures 6A, B**, ****p<0.0001 vs. control Sidak’s post-test). Neither BIX (500nM) nor UNC (1µM) alone impacted DCX levels in control OBSC (**Figure 5A**). However, both UNC (**Figures 6A, B**) and BIX (**Figures 6C, D**) prevented the ethanol-induced loss of neurogenesis, returning DCX to control levels (*##p<*0.01, *###p<*0.001 vs. EtOH, Sidak’s post-test). Regarding the effects of EVs, EtOH-EVs again reduced DCX (**Figure 6C**, *p<0.01 vs. pn-EVs, Sidak’s post-test). Further, like the effects of G9a inhibition on ethanol-treated OBSCs, BIX prevented the loss of neurogenesis caused by EtOH-EVs (**Figures 6C, D**, *##p<*0.01 vs. EtOH-EVs) while UNC showed a trend toward a reversal (p=0.18). In order to confirm that BIX and UNC effectively inhibit G9a/GLP complex activity at these concentrations, we measured H3K9me2 in EtOH-EV-treated OBSC +/- these inhibitors by IHC. A main effect of treatment was found (F_2,12_ = 22.9, *p*<0.0001) with both UNC (****p<0.0001, Sidak’s post-test) and BIX (***p<0.001) reducing H3K9me2 levels by 49% and 46% respectively (**Supplementary Figure 1B**), consistent with predictions from prior studies (61, 62). Thus, G9a/GLP complex activity facilitates the loss of hippocampal neurogenesis caused by EtOH and EtOH-EVs.

## 4 Discussion

**Figure 7:**
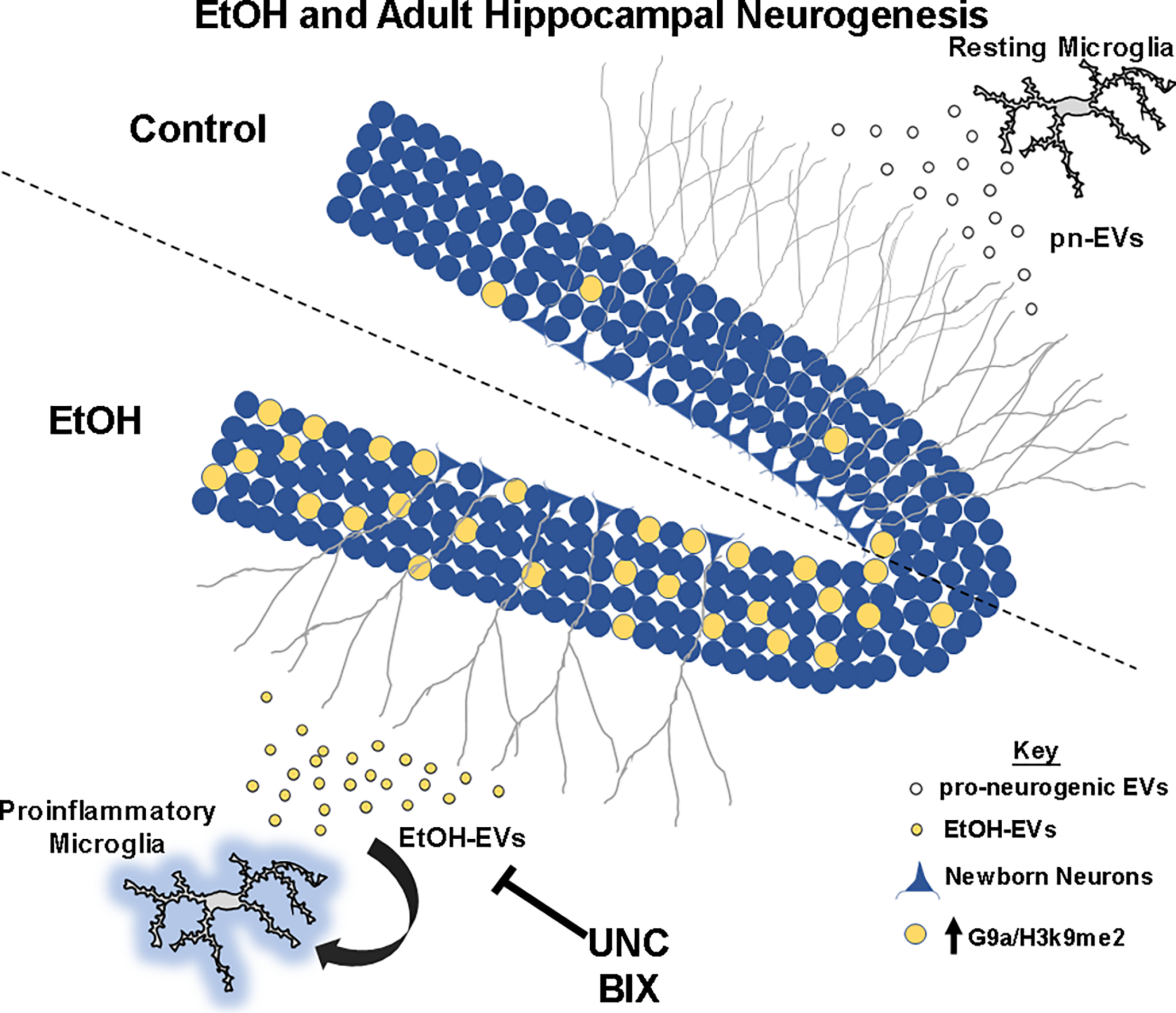
Summary of findings. At rest, microglia secrete pro-neurogenic EVs (pn-EVs) to promote neurogenesis (gray branches). Ethanol causes proinflammatory activation of microglia, with secretion of proinflammatory EtOH-EVs (Crews et al., 2021) now found to be enriched with G9a mRNA. EtOH induces G9a and H3K9me2 (yellow) in the neurogenic niche of the dentate gyrus to blunt neurogenesis (gray branches). Inhibition of G9a activity with UNC and BIX prevent loss of neurogenesis caused by EtOH and EtOH-EVs.

Adult hippocampal neurogenesis is an important neurological process that is inhibited by ethanol (5, 63). Chronic ethanol abuse causes dysfunction in hippocampal behaviors linked with AHN such as learning plasticity, memory, and mood (1–4, 64, 65). Thus, strategies that ameliorate the loss of AHN caused by alcohol could have a therapeutic benefit for behavioral dysfunction caused by alcohol abuse. Here, we report that extracellular vesicles from resting microglia promote adult hippocampal neurogenesis. However, EVs secreted in response to ethanol cause deficits in neurogenesis through a G9a/GLP-mediated mechanism (**Figure 7**). EtOH-EVs mimicked the effects of ethanol on neurogenesis and were enriched with G9a mRNA. Both ethanol and EtOH-EVs increased G9a levels in recipient cultures, and G9a inhibitors prevented the ethanol- and EtOH-EV-induced loss of hippocampal neurogenesis. This identifies a novel EV-epigenetic signaling system that could be targeted to improve cognitive and behavioral deficits associated with ethanol.

Both stressful and proinflammatory stimuli cause reductions in adult hippocampal neurogenesis (15–17). Microglia have recently been identified as regulators of AHN, with their phagocytic secretome increasing AHN (18, 19). In our previous report, we found that microglia induce EtOH-EVs that further promote the proinflammatory effects of ethanol (31). Here we find that resting microglia promote AHN through production of pro-neurogenic EVs. However, in the presence of ethanol, EtOH-EVs inhibit AHN through G9a. Here we find that G9a inhibitors blocked the EtOH- and EtOH-EV-induced loss of neurogenesis. Previously we reported the proinflammatory activity of EtOH-EVs was blocked by the anti-inflammatory inhibitor of HMGB1, GLY (31). However, GLY had no effect on G9a or H3K9me2 levels in these studies (not shown). This suggests that G9a-epigenetic signaling is either upstream or required in parallel with HMGB1 for proinflammatory gene activation to occur. Regarding adult hippocampal neurogenesis, a role of G9a/Ehmt2 has not been reported previously. However, other studies are consistent with this work, suggesting histone methylation represses AHN (66). A loss of the histone lysine-specific demethylase 1 (LSD1), which demethylates H3K9 (27) (resulting in increased methylation), was found to reduce neural stem cell proliferation (28), consistent with our finding. Global increases in H3K9 acetylation, the “opposing” epigenetic mark of H3K9me2, were found to increase differentiation of adult progenitors into neurons (10). Further, pro-neurogenic environmental enrichment reduced H3K9 methylation at the promoter of the neurogenesis promoting growth factor BDNF, increasing its expression (11). Thus, methylation of H3K9 in adulthood seems to inhibit AHN, though details regarding this regulation need to be explored further in future work. Though little is known about G9a regulation of adult hippocampal neuronal proliferation and differentiation, this “anti-neurogenic” role in adulthood seems to differ from its actions during embryonic development.

During embryogenesis, G9a promotes neuronal differentiation and suppresses expression of non-neuronal genes (12, 25, 26). However, evidence in adults suggests that H3K9me2 silences neuron lineage-specific genes in fully differentiated cholinergic neurons to promote a loss of the cholinergic phenotype after ethanol (30). Thus, the role of G9a appears to differ during embryonic and adult stages. This study is the first report to our knowledge that investigates the role of G9a in adult hippocampal neurogenesis. The impact of G9a on differentiation of other cell types in the adult hippocampus, however, remains unknown. It is possible that EtOH- or EtOH-EV-induced increases in G9a could promote differentiation of progenitors into other cell types, resulting in less neurogenesis. Further studies are needed to fully define the specific roles of G9a in proliferation and differentiation across cell types in the adult brain. It is important to note that the inhibitors of G9a signaling inhibit the G9a/GLP complex, also acting on GLP. Thus, the observed effects could be due to G9a or GLP. Our goal here is not to differentiate specifically between the roles GLP and G9a, rather to identify epigenetic mechanisms ex-vivo that can be moved *in vivo* in future work, with well-established inhibitors.

Though we found increased G9a mRNA in EtOH-EVs and induction of G9a in naïve OBSC by EtOH-EVs, it is unclear whether EtOH-EVs cause this increase through transfer of their mRNA. EVs can exert their influence in a variety of ways, many of which involve activation of immune pathways (67, 68). This can include transfer of contents to recipient cells (e.g., mRNAs, miRNAs), delivery of their contents to cell surface receptors (e.g., cytokines), or delivery of bioactive lipid species or damage-associated molecular pattern molecules (DAMPs, e.g., HMGB1). Recent studies have found that EVs play key roles in mediating multiple alcohol-related pathologies. In the periphery, this includes modulation of peripheral monocyte activity (69), promoting induction of monocyte chemoattractant protein-1 in hepatocytes (70), and induction of fibrotic genes in hepatic stellate cells through miRNA delivery (71). In the brain, microglial EVs promote hypothalamic neuronal death caused by ethanol during the embryonic period through delivery of the complement protein C1q (72) as well as neuronal death in adulthood through delivery of the miRNA let-7b (48). Astrocytes can also, in response to ethanol, secrete EVs that damage neurons involving a TLR4 neuro-immune pathway (73). An unexpected finding was that EVs from microglial-depleted cultures showed a trend toward a reduction of DCX in control OBSCs. It is possible that a loss of trophic factors in PLX-EVs or the presence of neurotoxic factors secreted in PLX-EVs mediate this, though future studies are needed to determine this. Nonetheless, EVs promote central and peripheral ethanol pathology, often through immune mechanisms. Consistent with this, we recently reported that EtOH-EVs induced by microglia seem to drive proinflammatory gene induction caused by ethanol (31). In that previous report, our studies suggested that MVs rather than exosomes mediate the pro-inflammatory signaling caused by EtOH-EVs. Here, we did not distinguish between the effects of MVs versus exosomes on AHN. Both MVs and exosomes are critical EVs, capable of modulating responses in recipient cells. Several factors known to regulate AHN have been found in exosomes (74), and MVs derived from mesenchymal stem cells are also able to promote neurogenesis (75). Thus, both MVs and exosomes could be involved in our observation, and should be investigated further in future work. This current work now implicates a role for EV modulation of epigenetic modifications to chromatin structure in both the induction of proinflammatory gene induction and loss of AHN caused by ethanol.

In summary, we found that microglia promote neurogenesis through secretion of pn-EVs and that EtOH-EVs inhibit adult hippocampal neurogenesis. The G9a/GLP complex is implicated in the loss of neurogenesis caused by ethanol and EtOH-EVs. In future work, we plan to investigate the ability of pn-EVs to reverse loss of AHN *in vivo.* The use of EVs as therapeutic agents could represent a promising biological medicine approach (76). Future studies will also investigate the gene targets of G9a in different cell types in brain, both *ex vivo* and *in vitro*, as well as the ability of G9a inhibitors to alter alcohol effects *in vivo.* Elucidating the targets of G9a and its function *in vivo* will determine if targeting this epigenetic pathway could be a beneficial therapeutic approach for mood and cognitive dysfunction in AUD.

## Data Availability Statement

The original contributions presented in the study are included in the article/**Supplementary Material**, further inquiries can be directed to the corresponding author.

## Ethics Statement

The animal study was reviewed and approved by UNC Institutional Animal Care and Use Committee (IACUC).

## Author Contributions

JZ and LGC drafted the text and figures. All authors contributed to the article and approved the submitted version.

## Funding

This research was funded by NIH, grant numbers: AA024829, AA028924, AA028599, AA020024, and The Bowles Center for Alcohol Studies.

## Conflict of Interest

The authors declare that the research was conducted in the absence of any commercial or financial relationships that could be construed as a potential conflict of interest.

## Publisher’s Note

All claims expressed in this article are solely those of the authors and do not necessarily represent those of their affiliated organizations, or those of the publisher, the editors and the reviewers. Any product that may be evaluated in this article, or claim that may be made by its manufacturer, is not guaranteed or endorsed by the publisher.

## Supplementary Material

Supplementary Figure 1 EtOH-EVs blunt IL-4 and UNC and BIX Inhibit G9a/GLP activity. **(A)** OBSCs were treated with either pn-EVs, or EtOH-EVs for 96 hours. pn-EVs caused an 82% increase in IL-4 gene expression (one-way ANOVA, F_2,6 _= 7.98, *p < *0.0001). **p < *0.05, ***p < *0.01, *****p < *0.0001, Sidak’s post*-*test. **(B)** OBSCs were treated with EtOH-EVs for 96 hours +/- UNC (1µM) or BIX (500nM). H3K9me2 was measured in slices by IHC. A main effect of treatment was found (One-way ANOVA, F_2,12_ = 22.9, *p*<0.0001) with UNC and BIX reducing H3K9me2 levels by 49% and 47% respectively, ****p < *0.001 *****p < *0.0001, Sidak’s post-test.
