# Supplementary figures and images for: Ethanol Induces Secretion of Proinflammatory Extracellular Vesicles That Inhibit Adult Hippocampal Neurogenesis Through G9a/GLP-Epigenetic Signaling
Source: Front Immunol. 2022 May 13;13:866073. doi: 10.3389/fimmu.2022.866073 (PMC9136051; doi:10.3389/fimmu.2022.866073)

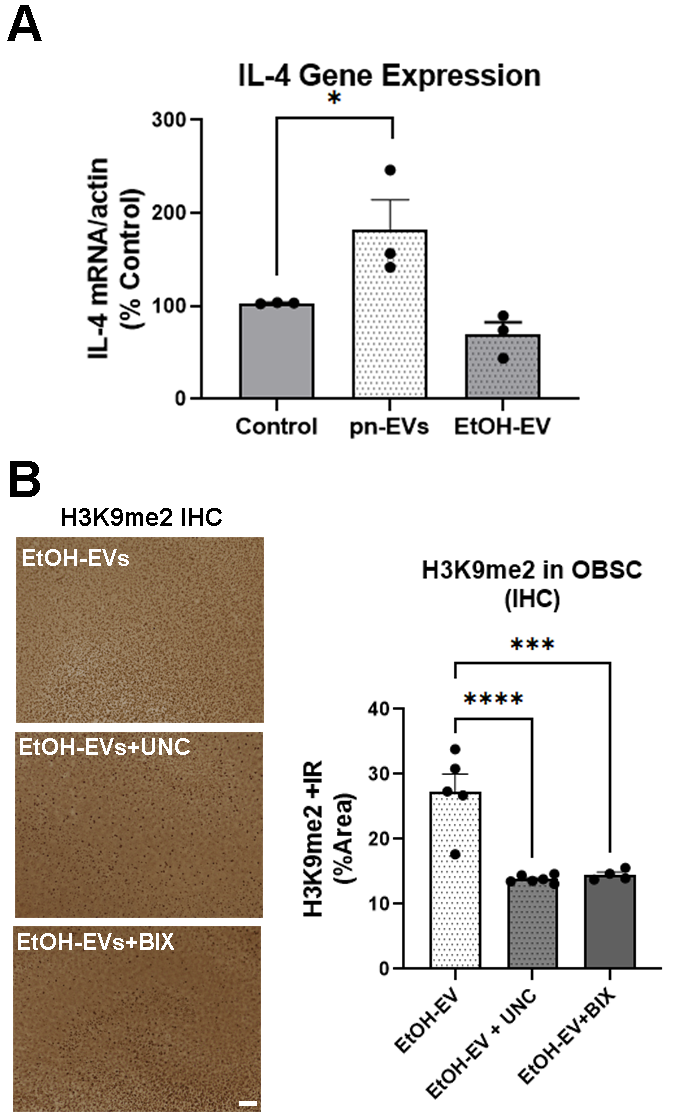

Supplement: Supplementary Figure 1 — EtOH-EVs blunt IL-4 and UNC and BIX Inhibit G9a/GLP activity. (A) OBSCs were treated with either pn-EVs, or EtOH-EVs for 96 hours. pn-EVs caused an 82% increase in IL-4 gene expression (one-way ANOVA, F2,6 = 7.98, p < 0.0001). *p < 0.05, **p < 0.01, ****p < 0.0001, Sidak’s post-test. (B) OBSCs were treated with EtOH-EVs for 96 hours +/- UNC (1µM) or BIX (500nM). H3K9me2 was measured in slices by IHC. A main effect of treatment was found (One-way ANOVA, F2,12 = 22.9, p<0.0001) with UNC and BIX reducing H3K9me2 levels by 49% and 47% respectively, ***p < 0.001 ****p < 0.0001, Sidak’s post-test. [file DataSheet_1.zip › Supp Figure 1-R2.tif]
